# Identifying and characterizing ideologically homogeneous clusters on Twitter and Parler during the 2020 election

**DOI:** 10.1371/journal.pone.0338318

**Published:** 2025-12-10

**Authors:** Daniel Verdear, Ashley Hemm, Zuoyu Tian, Sara El Oud, Sandra Kübler, John Funchion, Michelle Seelig, Amanda Diekman, Manohar Murthi, Kamal Premaratne, Neil F. Johnson, Stefan Wuchty

**Affiliations:** 1 Department of Computer Science, University of Miami, Coral Gables, Florida, United States of America; 2 Department of English, University of Miami, Coral Gables, Florida, United States of America; 3 Department of Linguistics, Indiana University, Bloomington, Indiana, United States of America; 4 Physics Department, George Washington University, Washington, District of Columbia, United States of America; 5 Department of Cinema and Interactive Media, University of Miami, Coral Gables, Florida, United States of America; 6 Department of Psychology, Indiana University, Bloomington, Indiana, United States of America; 7 Department of Electrical and Computer Engineering, University of Miami, Coral Gables, Florida, United States of America; 8 Institute for Data, Democracy and Politics, George Washington University, Washington, District of Columbia, United States of America; 9 Department of Biology, University of Miami, Coral Gables, Florida, United States of America; 10 Institute of Data Science and Computing, University of Miami, Coral Gables, Florida, United States of America; 11 Sylvester Comprehensive Cancer Center, University of Miami, Miami, Florida, United States of America; University of Exeter, UNITED KINGDOM OF GREAT BRITAIN AND NORTHERN IRELAND

## Abstract

During the 2020 U.S. presidential election cycle, a combination of public statements and social media posts cast doubt on the legitimacy of the election. These sentiments flowed through various social networks and eventually sparked the January 6th insurrection at the Capitol. Here, we analyze both the network-level and content-level data that made the #StopTheSteal movement so effective online. We use Louvain clustering and a novel homogeneity metric to identify the most ideologically homogeneous groups within the discussion on the mainstream social network Twitter and alternative social network Parler. We show that these ideologically homogeneous groups spread messages further than their ideologically diverse counterparts. Our results also differentiate between ideologically homogeneous left- and right-leaning groups by measuring the characteristics of their texts, finding that right-leaning texts are stylistically similar to worldbuilding language that can be found in conspiracy theory texts.

## Introduction

Over the course of the 2020 presidential campaign cycle, various figures on social media argued that the upcoming election would be fraught with rigging and fraud. Users leveraged features of social media sites to create ideologically homogeneous online environments, such as by creating hashtags like ’#StopTheSteal’ [1], allowing users to adopt a unified group identity [2] and form ’echo chambers’ [3–5]. Such echo chambers offer an environment where individuals are encouraged to socialize within their established connections [6,7]. Algorithmic recommendations, as implemented on various social media platforms, serve to further reinforce these constrains by pointing users toward similar information and opinions [4,8–13].

Previous work examining polarization on social media, often defined as a tendency toward both ideological homogeneity and extreme bias, uses a variety of methodologies to demonstrate that polarization and its psychological effects lead to distrust in media, which can in turn lead users to more fringe media sources [14]. Numerous studies show that social networks constructed using social media posts on a particular topic are often clearly divided into ideologically homogeneous groups e.g. [15,16]. These studies characterize polarization as a network phenomenon, focusing on patterns of user interaction over analysis of content.

In addition to network-level mechanisms that lead to polarization, several pieces of content-level information have been identified as impacting the popularity and virality of certain types of information on social media [17–19]. Previous research has demonstrated that the veracity of information is irrelevant to content spreading [20,21]. In fact, untrue information can cascade through a social network faster, suggesting a content bias where users are more likely to interact with content that has a negative emotional valence [22]. Additional work has pointed to the primary difference between mainstream news and fringe news being stylistic [23]. The framing of ideas in fringe news tends to rely on literary tropes and narrative forms that are distinct from the journalistic prose of factual reporting. Each of these studies highlighted the need to determine whether politically charged posts, such as those alleging voter fraud in the 2020 election, were more likely to use these highly viral tactics.

Here, we combine the analysis of the network-level information and content-level information that drive the formation of ideologically homogeneous clusters (IHCs) in the context of two social media platforms, Twitter and Parler. These two social media platforms were the most prominent public platforms used to broadcast the theory that the 2020 election was fraudulent. These platforms are functionally similar, but differ in the distribution with respect to political leaning of their users. Indeed, Parler was designed to be an alternative to Twitter for right-leaning users who were dissatisfied by Twitter’s hate speech and misinformation policies (this study was carried out prior to Twitter’s rebrand as X and the change of these policies) [24]. We compare these two platforms with respect to the network topologies that they encourage, how successful biased groups are at being heard, and the writing style choices of their users. We first characterize the differences between Twitter and Parler with respect to user distribution and interaction. Then we identify ideologically homogeneous clusters on each platform and characterize these groups with respect to topological measures and content. To this end, we define a novel metric that segments a social network graph into clusters and assigns each cluster a homogeneity score based on the ideological diversity of articles shared among its users. After identifying homogeneous clusters, we determine the ways these IHCs interact within the wider network and examine the engagement patterns between IHCs and less polarized groups. Finally, we determine what stylistic and linguistic characteristics differentiate the writings shared in IHCs from the average groups.

## Data collection and preprocessing

In the aftermath of the January 6, 2021 insurrection in the Capitol, Twitter carried out a widespread and rapid campaign to suspend accounts involved with the attack. Additionally, the controversial topic of election integrity claims led to an elevated number of voluntary tweet deletions. These two trends make post hoc data collection nearly impossible, as significant portions of the potential data became unavailable. Researchers, therefore, were forced to rely on datasets that were collected in real time so as to be resilient against these removals [25].

One such dataset is the VoterFraud2020 Twitter dataset published by Abilov et al. [26] that was collected in real time using the Twitter streaming API and a set of manually selected keywords and hashtags, such as #VoterFraud, #StolenBallots, and #StopTheSteal. This set of keywords and hashtags was programmatically expanded using word co-occurrence statistics. The VoterFraud2020 dataset contains 37M tweets collected between 10/23/2020 and 02/01/2021, which amounts to roughly 60% of all tweets published using a keyword or hashtag from their curated keyword and hashtag set. To facilitate our linguistic analyses, we augmented the VoterFraud2020 dataset with content-level data for tweets that contain an external link to a news or news-style article. For each such link, we collect the full text of the linked article as well as the name of the publishing website. These additional data allow us to calculate the bias of users and clusters in addition to the linguistic characteristics of clusters.

We used the VoterFraud2020 dataset to build a directed retweet network that captures the flow of information, originating in news and news-style articles, through the social network [15,27,28]. The tweet dataset was parsed to isolate retweets of tweets that contain an external link to a news article or outlet. A Twitter user may retweet a post from another user as a method of amplifying the original poster’s content. In this graph, each node represents a unique user, while each edge between two users represents a retweet. Edges are oriented in the direction of information flow, placing a directed edge from *j* to *i* if user *i* retweets content from user *j*.

Additionally, for edges that contain links to external content, we labeled each corresponding web domain (e.g. www.cnn.com) according to its editorial bias on a five-point scale from -2 (Left biased) to 2 (Right biased). Furthermore, retweets with links to the same domain were aggregated into a single edge by using the total count as an edge weight. We considered the bias of news articles as a function of their publishing domain by using data from Allsides.com and Mediabiasfactcheck.com, which are repositories of media bias classifications frequently used in the research literature [29,30]. These repositories label websites according to their story choice, factual veracity, and amount of partisan language used in the articles they publish.

In addition to the VoterFraud2020 dataset from Twitter, we collected data from the alternative social media platform Parler to compare our findings across platforms with different political landscapes. Though recent research is expanding the field of cross-platform analysis, these works are still uncommon because social media data are often difficult to directly compare, as different social media platforms naturally develop unique cultures that seem incomparable [31,32]. Among the research that does collect cross-platform data, most works use methods such as panel surveys to link their data at the user level rather than at the network level [33]. Other work specifically examines political candidates and public figures, whose cross-platform presence is easy to verify [34]. The literature on cross-platform online social networks is still growing, but has already established that different platforms encourage different groups structures and stylistic choices in text [35]. We advance the literature by describing the characteristics of these group structures, how the groups interact with the wider network, and the implications of different writing styles on Twitter and Parler.

Parler was designed to be a right-leaning counterpart to Twitter, sharing much of its core functionality, such as the ability of a user to repost - rather than retweet - another user’s posts. Therefore, a Parler repost network can be built in the style of the Twitter retweet networks described above and in the Methods section. Like the Twitter retweet graph, our Parler graph defines nodes as unique users and edges as reposts between users. Text and publishing website names are also collected from posts that include external links, and each publisher was given a bias rating on the [-2,2] scale. Posts regarding the 2020 election from Parler were gathered to match the timeframes in the Twitter data, collecting roughly 15M posts between 10/23/2020 and 01/11/2021. The data have an earlier end date, since Parler was taken offline by its web host following the Capitol insurrection [24].

## Methods

Each unique web domain that was linked to from a post in the dataset was classified on a five-point editorial bias scale (Left, Lean Left, Center, Lean Right, Right) using a combination of Allsides.com and Mediabiasfactcheck.com ratings. Tweets linking to any domains that were not present in either repository of political bias were discarded. This discarded data amounts to 29.135% of posts on Twitter and 14.901% of posts on Parler. These posts were discarded because they linked to other posts on the site, or to sources such as YouTube videos that would require subjectivity to classify. To facilitate numerical analysis, the five-point bias scale was transformed into numerical values in the range [–2,2]: –2 is Left, –1 is Lean Left, 0 is Center, 1 is Lean Right, and 2 is Right.

Retweet data from Twitter and Parler were filtered into chunks by date, and any retweets or reposts included in these chunks were parsed to create a set of directed graph edgelists corresponding to specific dates in our sampling period (10/23/20 – 1/11/21). The sampling period was trimmed to include only days with data from both platforms. A user’s potential edges are constrained by the days in which they were active on social media. To better capture these dynamics while maintaining robustness, we aggregate our data into a set of discrete time windows. Our total retweet network graph, *G*(*V*,*E*) where *V* is the set of nodes and *E* is the set of edges, was split into daily timesteps by filtering the date and time of each post or retweet. Then, these daily timesteps were aggregated into windows of 10 days, $G_{t}(V_{t},E_{t})$ for a start date *t*. The new graph *G*_*t*_ contains all users and retweet relationships that occur in the data starting on day *t* and extending through day *t* + 9. Each subsequent time window shifts with a stride length of 1, so by this convention, timestep $G_{t+1}(V_{t+1},E_{t+1})$ uses the retweets starting on day *t* + 1 and ending on day *t* + 10.

### Community detection and labeling

To ensure that our data contain viable communities, these time window graphs were used to calculate the global clustering coefficient, a metric that quantifies the density of connections among triads in the graph as a value [0,1]. Social network graphs, or in our case, graphs that model genuine social interaction, tend to have higher clustering coefficients than randomly chosen graphs of the same size [36]. For each time window, the clustering coefficient of its nodes was calculated according to:

$$c_{u}=\frac{T(u)}{2(deg(u)(deg(u)-1)-2deg^{rec}(u))}$$ (1)

where *T*(*u*) is the number of directed triangles through node *u*, *deg*(*u*) is the combined number of in- and out-edges, and *deg*^*rec*^ is the number of reciprocal edge pairs containing *u*. The global clustering coefficient is then taken by averaging these values over the entire graph. We find that the average clustering coefficient is 2.9298E-2 for our Twitter data and 5.2375E-2 for our Parler data. Each time window is then compared to a random graph of similar size. We generated a random Erdős-Rényi graph with an identical number of nodes and edges, then repeated the clustering coefficient calculation [37,38]. An Erdős-Rényi graph is generated by specifying a number of nodes and of edges, then a graph is randomly chosen from the set of unique graphs with that amount of nodes and edges. We find that the average clustering coefficient is 1.1099E-5 for the random Twitter-sized graphs and 2.7381E-4 for the random Parler-sized graphs. This multiple order of magnitude improvement in clustering coefficient over the random graphs suggests that running community detection will lead to viable communities. We additionally compare our networks, which are comprised primarily of political discussion posts, to the non-political social networks from the Higgs-Twitter dataset [39]. The Higgs-Twitter retweet network has a clustering coefficient of 1.5600E-2, which is on the same order of magnitude as our Twitter and Parler data. This is an additional confirmation that our data retains the characteristics of a typical online social network.

At each time window, the Louvain algorithm was run to segment the data into clusters, a procedure which is both deterministic and efficient on large graphs. This is a greedy algorithm that assigns nodes to a cluster with high intra-cluster density by optimizing the modularity of each cluster, or the ratio of within-cluster edges compared to between-cluster edges, according to the following equation:

$$Q=\frac{1}{2m}\sum_{i,j}\left(A_{ij}-\gamma \frac{k_{i}k_{j}}{2m}\right)\delta (c_{i},c_{j})$$ (2)

where *m* is the number of edges in the graph, *A* is the adjacency matrix, *k*_*i*_ is the degree of node *i*, *γ* is a parameter set to 1, and $\delta (c_{i},c_{j})$ is a Kronecker delta function that returns 1 if nodes *i* and *j* are in the same community and 0 otherwise. We find that the average modularity is 0.7057 for our Twitter data and 0.7281 for our Parler data. These results demonstrate that our networks contain well-defined communities roughly in line with the modularity findings of social networks from the literature [40].

For each of the resulting communities, we calculate several cluster-level values that are used in the analyses of clusters. It is at this level of the Louvain cluster that we define the ideological homogeneity of groups in the network. In addition to this value, we calculate the average bias of the cluster’s articles, the number of intra-cluster and inter-cluster retweets or reposts generated by the cluster’s articles, and the size of the cluster. We also calculate the values of genre perplexity from the cluster’s articles, described below.

### Ideological homogeneity metric

Previous research has shown that an individual’s media diet is typically diversified [41], but follow-up work in psychology argues that people give higher regard to media that they have actively interacted with [42]. Therefore, a retweet network captures these more highly regarded pieces of media, and our metric captures the diversity of these more salient sets of information. We are careful to refer to these retweeted articles using the ideologically neutral term *highly regarded media* as the use of retweets as a method to measure endorsement is still subject to debate in the literature. Previous works show that users often retweet articles for the purpose of debunking or arguing against its contents [43,44]. Whatever their intentions, a user repeatedly retweeting biased sources serves to amplify those sources, and the network effects are functionally similar [45,46].

To measure the extent to which users in our graph have balanced media interactions, we propose a novel metric for measuring the ideological homogeneity of a subset of users in a social network. The metric develops on the graph theoretic concept of the clustering coefficient and the information theoretic concept of entropy. We quantify ideological homogeneity as a function of the diversity of a user’s set of news sources, measured by calculating a variation of entropy on that source set. The value is then weighted based on the number of users in the cluster. Each of our clusters consist of edges that represent information flowing from a producer (the source node) to a consumer (the target node), and each edge has a bias label corresponding to the specific web domain it links to. Our metric measures the extent to which the total set of consumers in a graph have balanced media interactions. Our metric is also unique from other models, such as latent ideology detection, because our metric does not rely on subjective classification of text [47]. Current state of the art methods for latent ideology detection in text add significant complexity to data annotation. Such procedures are typically only accurate on domains where there is richly labeled training data, and those domains are often too specific to be useful generally [48,49].

For a group *G*(*V*,*E*), the consumer set C⊆V is defined as the set of all target nodes for the edges of *G*. For a given consumer node, we define the label set *L* as the set of bias values for each article that consumer node has retweeted. Thus, for a group *G* with consumer set *C*, and label set *L*, the ideological homogeneity of *G* is calculated as:

$$I(C)=\frac{\sum_{v_{i}\in C}(1-H(v_{i}))}{\left|C\right|}$$ (3)

$$H(v_{i})=-\sum_{l\in L}p(l,v_{i})\log_{2}(p(l,v_{i}))$$ (4)

where $H(v_{i})$ is the entropy of the set of bias labels for all edges into node $v_{i}$. Additionally, the probability term $p(l,v_{i})$ corresponds to the fraction of the edges of node $v_{i}$ which have bias label *l*. As defined in Eq 3, this metric quantifies the extent to which *G* has ideologically homogeneous interactions as a value in [0,1] [50]. When each potential bias label is present in equal proportions in the label set, the entropy $H(v_{i})$ of the label set is maximized at 1. In this case, the $\left(1\;-\;H(v_{i})\right)$ term will resolve to 0, since a cluster that shares articles of all bias labels in equal proportions is not ideologically homogeneous. Conversely, if a cluster shares articles from only one bias label, the entropy of the label set is minimized at 0; therefore, the $\left(1\;-\;H(v_{i})\right)$ term resolves to 1 since this is an ideologically homogeneous cluster. The equation then weights that value based on the number of users in the cluster. *I*(*C*) will gradually decrease along the [0,1] range as more ideological diversity is added to users’ articles. For each time window, each Louvain cluster was assigned a ideological homogeneity value according to Eq 3. Previous work has identified that upwards of 20% of the US population is polarized, and follow-up work uses this upper quintile to examine membership in polarized bubbles online [51–53]. Therefore, for both Twitter and Parler, we define ideologically homogeneous clusters (IHCs) as the set of clusters in the top 20% with respect to this value. To further test the robustness of our results, we repeated the graph topology experiments with two additional cutoff values (15% and 25%). These results do not differ significantly from the 20% cutoff, so we continue using the 20% as our baseline IHC cutoff value. The full distribution of ideological homogeneity values and the IHC cutoff for both platforms are visualized in S3 Fig.

### Article spread using retweets

After dividing our data into clusters and labeling the most homogeneous clusters as IHCs, we measure the extent to which clusters are able to spread their message throughout the network. To do so in a way that uses our available data, we measure the retweets garnered by articles posted by members of a cluster. Measuring impact this way requires careful interpretation because the retweets on an article do not necessarily correlate with overall acceptance of its contents. Regardless of the level of acceptance, a cluster’s ability to gather retweets from the wider network demonstrates its ability to set the tone of online discourse. We compare IHCs on both platforms to less homogeneous clusters in their ability to gather retweets.

To ensure that comparisons are made between similarly sized clusters, we group all clusters in the full dataset into 40 bins as a function of their size. 40 bins were chosen because our most frequent cluster size (3) makes it so that 40 is the largest number of bins that fits all clusters of a certain size into the same bin. The bins generated by this procedure are described in detail in S2 Table. After controlling for cluster size, we calculate the average number of retweets per user in IHCs and the retweets per user in all other clusters. The average retweet value *R* for a set of clusters 𝕏 is defined as

$$R(𝕏)=\sum_{i\in 𝕏}\frac{RTs(i)}{size(i)}(\frac{1}{\left\|𝕏\right\|}),$$ (5)

where *i* is an individual cluster, *RTs*(*i*) is the total retweets for posts in that cluster, and *size*(*i*) is the number of users in that cluster.

### Genre perplexity

In addition to the graph metrics, each cluster is assigned a score to quantify the extent to which its articles use language indicative of certain literary genres. A trigram language model was trained using the SRI Language Modeling toolkit for each of 14 genres available in the BookCorpus dataset [54,55]. The list of genres is included as S2 Table. Each language model was trained on the BookCorpus data corresponding to the specific genre of writing, and the trained model is a probability distribution of trigrams contained in the genre writings [56]. We use an n-gram based language model rather than a neural one since we need the model to focus on lexical cues and local context, which we cannot guarantee in a neural model. Additionally, since we can train the n-gram model on solely on the BookCorpus, we avoid data leakage.

For each pair of article and genre, the genre language model is used to predict the text of the article. The predictions are reported as perplexity scores and averaged at the article level. Perplexity scores measure the amount of surprise toward seeing a given sequence of words, and they are often used as a similarity metric to determine similarity between texts; lower perplexity means the given text is more similar to the training corpus [57]. In our experiment, this implies that the article’s text is similar to the writing style indicative of the genre. We intentionally use pre-neural language models in order to avoid data leakage and to focus on local, morph-syntactic similarities. With other language model architectures, it is unclear on what linguistic level the similarity is determined.

We then compare each cluster’s average perplexity to the dataset average by measuring the Z-score as in Eq 6. For a cluster *c*, let *x*_*c*,*g*_ be the average perplexity value for *c*’s articles with respect to genre *g*. This cluster’s perplexity is then compared to the dataset average for all articles shared with respect to that genre, specified by the mean and standard deviation $\mu_{g}$ and $\sigma_{g}$.

$$Z=\frac{x_{c,g}-\mu_{g}}{\sigma_{g}}$$ (6)

## Results

Although Twitter and Parler share similar functionality, prior work has shown that the two platforms have vastly different user demographics [58]. Our first analysis demonstrates how these disparate demographics manifest within our dataset of posts about the 2020 election. We calculated the average leaning of each user in our Twitter and Parler datasets by taking the arithmetic mean of the bias values of all websites whose articles are posted or retweeted by the user. For a user *u*, we define *a*_*u*_ as the total number of articles user *u* posts or retweets and calculate the average of each article’s bias, *B*(*p*) for an article *p*, according to

$$x(u)=\frac{\sum_{j=1}^{a_{u}}B(p_{j})}{a_{u}}.$$ (7)

**Fig 1:**
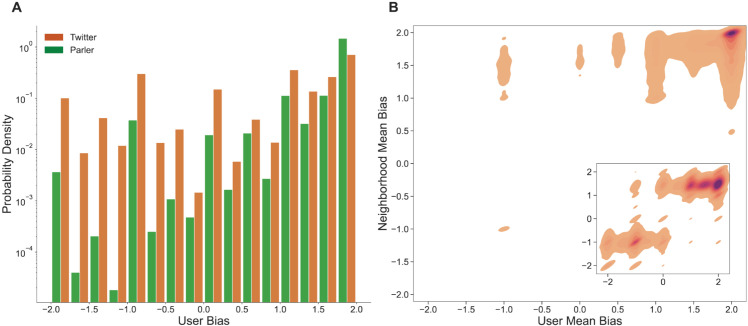
User bias distributions on Twitter and Parler. (A) Each bin represents a 0.25-point interval of average user bias, and the height is the probability density of users in that bin. Twitter contains similar proportions of left- and right-leaning users, while Parler is predominantly right-leaning. (B) Contour plots of user average bias with respect to the average bias of their one-hop neighborhood. In both plots, –2 refers to the most left-leaning, and 2 to the most right-leaning users. Additionally, darker shades represent a higher density of interactions. Two homophilic clusters characterize Twitter users, while most users’ neighborhoods are right-leaning on Parler.

In Fig 1A, we observe that Twitter contains a balanced population of left- and right-leaning users, while the population on Parler skews to the right. For our experiments, we define a user to be left-leaning if their average leaning according to Eq 7 is less than –0.5 and right-leaning if their average leaning is greater than 0.5.

We then examined our Twitter and Parler social networks by characterizing their interaction trends [4]. For each user *u*, we calculated the average leaning of their immediate neighborhood by averaging the leanings of all articles shared by users that are adjacent to *u* in the underlying networks. Similarly to the user-level calculation, we define *an*_*u*_ as the total number of articles posted or retweeted by the users in the one-hop neighborhood of *u* with the average bias being calculated as

$$x(u)=\frac{\sum_{j=1}^{an_{u}}B(p_{j})}{an_{u}}.$$ (8)

**Fig 2:**
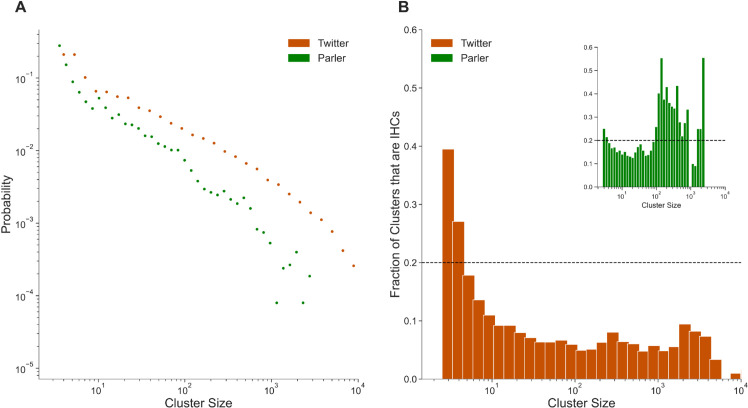
Characteristics of ideologically homogeneous groups. (A) The probability distribution of cluster sizes on both social networks exhibit exponential decay, indicative of the prevalence of smaller clusters in our results. (B) Percentage of clusters that are ideologically homogeneous (IHC) with respect to cluster size. We define IHCs as the top 20% most homogeneous clusters (dotted line) and observe that Twitter IHCs are concentrated in smaller clusters, while larger groups are more likely to be homogeneous on Parler (inset).

In Fig 1B, we observe two homophilic clusters of left- and right-leaning users on Twitter (inset), while Parler is dominated by right-leaning users absent any left leaning population. Notably, these homophilic groups are characterized by dense interaction within themselves, with very few cross-population edges. We see this in Fig 1B as empty space in the top-left and bottom-right quadrants. For the upcoming analyses of ideological homogeneity, we use a more sophisticated community detection procedure than simple one-hop neighborhoods. To produce communities that favor groups with high intra-cluster density, we applied the Louvain clustering algorithm on each of our 10-day time windows [59]. In particular, the Louvain algorithm divides a network graph into clusters by optimizing the ratio of within-cluster edges to between-cluster edges. In the probability density function in Fig 2A, we observe that the size distributions of clusters on both Twitter and Parler decay exponentially, indicating a prevalence of small over larger clusters in both networks as would be expected in a typical social network.

Before conducting experiments on our individual timestep clustering results, we must ensure that the clustering is stable over time. The clustering results could be dominated by noise, and in such a situation, a user’s neighborhood may be split into clusters randomly at each timestep. We measure the stability using the Adjusted Rand Index, which quantifies the number of pairs which are labeled consistently across two consecutive clustering results [60]. The clustering is found to be sufficiently stable for further analysis (S1 and S2 Figs).

### Characterizing the ideologically homogeneous clusters

Based on these clusters, we calculate the ideological homogeneity of the set of clusters in each time window. The most homogeneous quintile are then labeled as ideologically homogeneous clusters, or IHCs. To uncover where in our data the most polarized groups are overrepresented, we first examined ideological homogeneity with respect to the cluster size (Fig 2B). We find that on Twitter the smaller sized clusters are enriched with IHCs, containing much higher proportions than are attributable to random chance (indicated by a horizontal dashed line at 0.2). This is expected behavior, as smaller groups are more likely to be homogeneous than larger groups. Smaller groups minimize the denominator of our ideological homogeneity metric and have fewer opportunities to diversify their information sources [61,62]. Clusters of size 3 through 7 have statistically significant enrichment of IHCs, which was verified using permutation tests (*p* < 0.01).

On Parler, IHCs are distributed differently than on Twitter as the larger clusters with size 100 through 1,000 users are significantly enriched with IHCs (inset, Fig 2B). We used permutation tests to find that clusters between size 99 and 965 are significantly more likely to be IHCs than random chance would indicate (horizontal dashed line at 0.2; *p* < 0.01). One potential explanation for this divergence is based on Parler’s largest growth periods being driven by conservative commentators publicly promoting the platform, and subsequent bursts of users joining Parler are dominated by followers of these commentators [24]. Such a trend potentially led to Parler being more monolithic in its cluster structure, as well as its bias.

### Ideologically homogeneous clusters spread articles more widely

Having described the ideologically homogeneous subpopulation on each platform as a function of cluster size, we subsequently determine the effect they have on the wider social networks they participate in. If the most homogeneous clusters post content that is incendiary but fails to resonate with users, their real impact is negligible. On Twitter and Parler, users can retweet or repost another user’s content as a means to broadcast to their followers. This functionality allows information to potentially cascade through a network rapidly. Thus, the amount of retweets a group can gather on its posts is a useful proxy for understanding which groups are resonating best with the user base. For this reason, we examined the retweet and repost distribution of ideologically homogeneous clusters relative to the less homogeneous clusters with respect to three retweet metrics.

**Fig 3:**
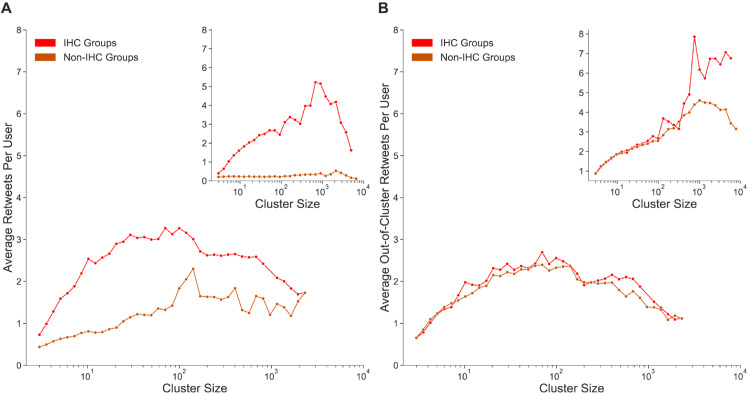
Retweet characteristics of IHCs. (A) Retweet spread ratio as a function of cluster size on Parler and Twitter (inset). Retweet spread ratio is defined as the ratio of retweets per user in IHCs and retweets per user in all other clusters. For all cluster sizes, the IHCs have significantly higher retweet spread than similarly sized but less homogeneous clusters. On Twitter, the IHCs are much more effective at gathering reposts when compared to Parler. (B) Inter-cluster spread ratio as a function of cluster size for Parler groups and Twitter groups (inset). Inter-cluster spread ratio was calculated as the ratio of the number of edges from a cluster to another unique cluster and the number of users in that cluster. The results show that users on Parler tend to refrain from interacting outside of their primary clusters, regardless of the ideological homogeneity value. On Twitter, IHC groups are more effective at gathering out of cluster retweets, a differential that grows for larger clusters.

Our first, and simplest, measure of a group’s message spreading through retweets is the absolute number of retweets gathered by IHCs. Fig 3 compares the average retweets per user in IHCs to non-IHC groups for 40 cluster size bins. In Fig 3A, we observe that in every cluster bin IHCs receive a higher number of retweets or reposts, a finding that was confirmed using a Welch’s t-test (*p* < 0.01). Interestingly, we find that the largest differences occur for cluster sizes with fewer IHCs. In addition to the analysis of raw retweets, which may include factors like retweets from out-of-sample users or a high number of retweets coming from within a post’s cluster, we directly examine the extent to which a cluster’s posts entangled users from other clusters. Notably, such calculation offers insight into how effective IHCs are at spreading messages outside of their immediate contacts. Similarly to the previous analysis, we control for cluster size by segmenting the data into 40 bins and count for each cluster the number of edges to other clusters. These values are averaged on a per user basis. In Fig 3B we observe a divergence between the two platforms, where both IHCs and less-homogeneous clusters have similar inter-cluster retweet values that tend to be low on Parler. Such observations suggest that users of the alternative social media platform tend to confine themselves to their immediate contacts regardless of their ideological homogeneity value. On Twitter, inter-cluster retweet values correlate with cluster size, and larger clusters reach more external clusters. Additionally, IHCs larger than 500 users reach significantly more external clusters (Welch’s t-test: *p* < 0.01).

Finally, we examine the participation of IHCs in the edge boundary on Twitter. For this purpose, we consider the edge boundary of a retweet graph as the set of edges that connect a right-leaning cluster to a left-leaning cluster. We consider a cluster to be left-leaning if the average leaning of its users is less than –0.5 and right-leaning if the average leaning of its users is greater than 0.5. For each time window, we calculate the fraction of edges along the boundary that include at least one user that belongs to an ideologically homogeneous cluster. On average, across each time step in the Twitter data, only 2.87% of boundary edges contain a user from an IHC. If edges in the graph were assigned randomly, we would expect 15.05% of boundary edges to contain users from an IHC, suggesting that ideologically homogeneous clusters very rarely interact with differently biased groups (S3 Fig).

### Article texts reveal a divergence in language use

Previous works have argued that a central theme in conspiracy theory texts is the frequent use of worldbuilding language [63–65]. Conspiracy theory texts, as a general template, do not attempt to logically persuade the reader to reject the mainstream view and adopt their own worldview. Instead, they offer a characterization of the world where their views are portrayed as a natural consequence [66,67]. Therefore, we also measure the linguistic and stylistic characteristics of the articles that are shared in clusters with high ideological homogeneity and higher likelihood to spread their messages through the network. From these insights, we determine whether shared right-biased articles supportive of the voter fraud conspiracy theory have similarity to worldbuilding language as suggested in previous work.

Detecting a high-level concept such as worldbuilding is not a simple task. To accomplish this task, we first had subject matter experts assign genre labels to each corpus based on its texts’ narrative and formal features. The writing genres were divided into three categorically similar groups, Worldbuilding and Speculative Fiction genres, Hybrid genres, and Non-Speculative genres. These classifications can be seen in Table 1. The Worldbuilding and Speculative Fiction genres represent fiction writing that dedicates significant time to characterizing its world, while the Non-Speculative genres contain minimal amounts of text describing the world and other contextual factors. The Hybrid genres contain writing along the spectrum of worldbuilding that contains elements of speculative writing, but not enough for texts to be considered full Speculative Fiction. Detailed descriptions of the 14 genres are included as S2 Table.

**Table 1:** Genres categorized by theme.

Generic Category	Genre
Worldbuilding/Speculative	Alternative History
	Fantasy
	Dystopia
	Children’s Adventure
	Science Fiction
	Eschatology
	Spiritual
Hybrid	Crime
	Epiphanic Writing
	Morality Writing
	Political Treatises
Non-Speculative	Action and Adventure
	LGBT Fiction
	Activism

We trained a trigram language model on each of these 14 genres to learn the probability distributions of words in each genre. We then passed each article shared within our Twitter and Parler datasets through these language models, assigning a perplexity score to quantify its similarity to each of the 14 writing genres. Such a perplexity score measures how well the language model is able to predict the next word of a text using the three previous words and its genre-specific training data. Lower perplexity scores indicate that a text is easy to predict using the training data, which we infer implies the text is stylistically closer to the training genre. Notably, each cluster is assigned a score to roughly quantify the extent to which its articles use language indicative of certain literary genres.

To show that our genre experiment methodology produces legitimate results, we run a baseline experiment using Facebook data from pro-vaccine and anti-vaccine groups [16]. The results show that the anti-vaccine groups post content that is most similar to the speculative fiction genres and several hybrid generes (S4 Fig). These genres, specifically the Epiphanic and Eschatology genres, point to a narrative describing an awakening to the reality of corrupt activity hidden from view, a result consistent with descriptions of conspiracy narratives. Comparatively, the pro-vaccine texts are most similar to the Political Treatises hybrid genre and the Activism non-speculative genre, both indicative of lower amounts of worldbuilding.

For our subsequent experiment on the Twitter and Parler data, we calculate the average perplexity score across each dataset for the 14 genres we investigated. The perplexity scores measure the word prediction entropy of a language model trained on writings from a certain genre; lower values of perplexity indicate that a text is closer to that genre in terms of style and word choice.

**Fig 4:**
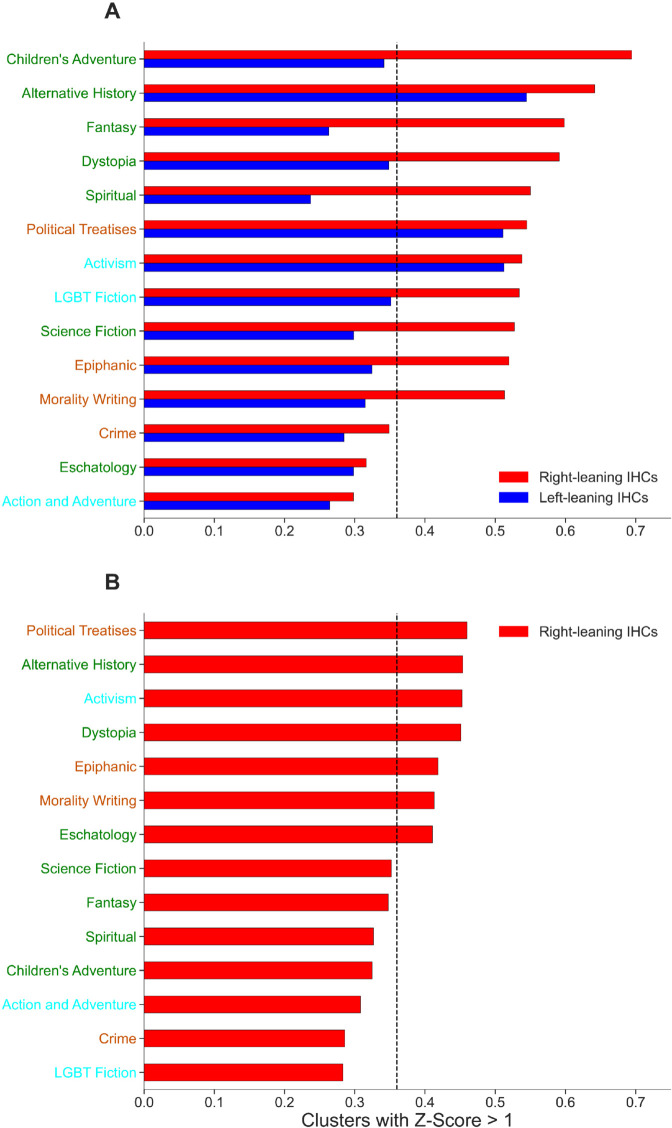
Fraction of all IHCs that are at least one standard deviation below the average value with respect to writing genre. (A) On Twitter, left-leaning groups post content that have high similarity to the Alternative History genre and the Activism Non-Speculative genre, while the right leaning groups posts are most similar to both Speculative Fiction and Hybrid genres. Conversely, on Parler (B), the right-leaning groups have significant similarity to the Hybrid genres as well as the Alternative History and Activism genres indicative of the election context. The dotted line indicates the expected, random distribution of genres.

Our experiment seeks to determine whether IHCs in our data are likely to show higher similarity to the Worldbuilding genres than expected by chance. We consider an IHC to be *highly similar* to a genre if it has a Z-score below –1, indicating that it is more than one standard deviation below the genre mean. Each IHC was then given a binary label to signify whether its texts are highly similar to each genre. These binary labels are used as the input to a permutation test, the result of which identifies the genres that are enriched with IHCs more than would be predicted by random chance. Fig 4 quantifies this random chance cutoff as a vertical dashed line and demonstrates for both platforms which genres’ highly similar clusters are enriched with IHCs, visualized as bars crossing this cutoff, more than random chance would predict. To further increase the precision of our experiment, we split the set of IHCs on Twitter and Parler into subsets of left-leaning IHCs and right-leaning IHCs. We consider a cluster to be left-leaning if the average leaning of its users is less than –0.5 and right-leaning if the average leaning of its users is greater than 0.5.

On Twitter (Fig 4A), we find that content that the right-leaning IHCs post have above average similarity to most of the speculative fiction genres and hybrid genres, as well as the Activism genre. When these ideologically homogeneous election skeptic groups are broadcasting to a wider mainstream audience, they rely on worldbuilding to frame their worldview as a compelling call-to-action. In particular, there exists a mixture of worldbuilding narratives and factual reporting among the articles shared in these right-leaning IHCs. In turn, left-leaning IHCs are most similar to the Alternative History speculative genre and the Activism and Political Treatise genres. We suppose that Alternative History and Political Treatises are genres common to all IHCs because these genres are indicative of the political subject matter being discussed. Otherwise, we find that on Twitter, the ideologically homogeneous right-leaning groups rely more on using worldbuilding language to convert users on the more popular platform to their worldview.

The results for right-leaning clusters on Parler (Fig 4B), paint a different picture. The Alternative History and Dystopia speculative fiction genres are significantly represented as they were on Twitter, but these were the only worldbuilding genres with significant similarity to articles shared among Parler IHCs. All other significantly similar genres may contain conspiracy tropes but do not contain distinct worldbuilding language. Due to the majority of Parler IHCs being right-leaning, the left-leaning IHCs which do exist on the platform showed no noteworthy genre similarities. These results indicate that, when among users with similar views, or more specifically among users that do not need to be convinced to question the election, the ideologically homogeneous election skeptic groups do not follow worldbuilding forms as strictly.

## Conclusion

In their totality, our findings about ideological homogeneity add depth to previous research on echo chambers and online polarization. Our findings also expand the literature on cross-platform social media events by demonstrating how users on different platforms discuss the same event. We defined an ideologically homogeneous cluster as a group on social media where external information, typically articles or texts, comes from websites that share similar biases. In the context of discussion about the 2020 US presidential election, these homogeneous clusters tend to be smaller on Twitter and larger on Parler. Other work has suggested that, in order to evade moderation, polarized groups on a mainstream platform such as Twitter adapt to become smaller and more numerous [68]. There is no need for such tactics on a platform like Parler, which prioritizes its own lax moderation policy. Our results demonstrate that the perceived homogeneity of a platform as a whole leads to distinct changes in the network topology of groups of users.

Much like previous research on false or polarized news, we find that ideologically homogeneous clusters are likely to spread messages further than their more balanced counterparts. On both platforms, posts from ideologically homogeneous clusters receive more interactions than posts from other clusters when controlling for cluster size. These groups are shown to have more total retweets as well as slightly more retweets from outside of their own cluster. The edge boundary of our graphs, or the posts that cross from left- to right-biased groups, are unlikely to contain posts from these ideologically homogeneous clusters. These groups can take various topological forms depending on the platform they exist on, and they present information that is better at gaining traction, which poses a threat to informed discourse on social media platforms.

A new body of research is considering conspiracy theories and their supportive texts as a literary phenomenon rather than a persuasive argument. As shown by Seelig et al., conspiracy theory texts rely on narrative tropes and worldbuilding language to frame themselves as an account of societal decline that can only be stopped by the action of the reader [66,67]. We expanded upon previous works that measured the content-level differences using black box methods such as toxicity detection by directly measuring the presence of this style of worldbuilding language among clusters on each platform. We find that on Twitter, ideologically homogeneous clusters were significantly more likely to post articles that shared stylistic elements with worldbuilding genres. These articles were often posted among right-leaning users who were most vulnerable to partisan-motivated reasoning following an election loss [69]. Interestingly, users on the much more homogeneous platform Parler shared articles with far less worldbuilding language, pointing to a potential linguistic difference among platforms of differing homogeneity. However, our perplexity results only give a baseline indication of similarity. Further study of polarized news sources, either using more sophisticated language modeling or qualitative analysis, is required to make deeper conclusions about worldbuilding language in polarized news.

Further study of political events on social media is necessary to determine whether the 2020 #StopTheSteal movement is unique in its topological and stylistic elements or if any of these results can generalize to other political calls-to-action. Most current studies only examine a small number of real-world events, but our cross-platform results show that even the same event can manifest in different ways on different platforms. Future work should focus on determining whether platforms respond similarly to other real-world events, meaning the behavior is characteristic to each social media platform. Otherwise, each major event must be examined as unique and each platform’s response must also be examined as unique. New results that potentially demonstrate similarities can point the field past understanding the problems of echo chambers and into determining effective countermeasures.

## Supporting information

S1 FigCluster stability over time.Before conducting experiments on our individual timestep clustering results, we must ensure that the clustering is stable over time. The clustering results could be dominated by noise and difficult to draw results from; in such a situation, a user’s neighborhood may be split into clusters randomly at each timestep rather than remaining stable. We measure this stability using the Adjusted Rand Index, which quantifies the number of pairs which are labeled consistently across two clustering results [60]. The Adjusted Rand Index (ARI) is calculated using:
$$ARI=\frac{RI-E[RI]}{1-E[RI]}$$
(9)
$$RI=\frac{a+b}{C_{n}}$$
(10)where *RI* is the equation for unadjusted Rand Index, *E*[*RI*] is the expected value for the unadjusted Rand Index, *a* is the number of pairs that are in the same set in both clustering results, *b* is the number of pairs that are in different sets in both clustering results, and *C*_*n*_ is the total number of pairs in the data.The plots of ARI over time for Twitter are shown in S1 Fig. (A) and (B) and for Parler are shown in S1 Fig (C) and (D). In general, both plots demonstrate stable clustering over time, with slight daily noise caused by users joining the graph or genuinely moving clusters. An ARI of 0 signifies clustering results that are distributed uniformly at random, so both social networks having an ARI within the [0.4, 0.7] range means cluster stability is relatively high. Both platforms have a steep decline in ARI early on, which is attributable to a large number of new users entering the discussion. Twitter’s decline occurs within the first few days of the data, while Parler’s decline occurs closer to the date of the actual election; this is likely an artifact of Twitter being an established platform and Parler being a smaller platform reliant on new signups to grow its network. To visualize this relationship with the size of each day’s graph, the normalized size of each daily retweet graph is plotted alongside the ARI.After a long period of relative stability in both platforms, we see a divergence between the platforms in cluster stability post-January 6. Parler stability steeply increases, pointing to a hardening of the cluster structure. On the other hand, Twitter stability gradually decreases as moderation removes nodes from the graph.(TIFF)

S2 Fig Ideological homogeneity score distributions. In the main text, we define IHCs as the top 20% of clusters according to the ideological homogeneity metric, and S2 Fig. visualizes the cumulative distribution of the ideological homogeneity metric for Twitter and Parler. The minimum value to be considered an IHC on Twitter is 0.375 (with an IHC mean of 0.607). To be considered an IHC on Parler, the minimum value is 0.75 (with an IHC mean of 0.892).
In addition to showing that IHCs are more homogeneous on Parler than on Twitter, the distribution shows that clusters in general are more homogeneous on Parler than Twitter. The median ideological homogeneity on Twitter is approximately 0.2, while the median on Parler is approximately 0.5. The systemic differences between these two social media platforms, discussed in the main text, are the likely cause of these disparate distributions.
(TIFF)

S1 Table Cluster bin sizes and counts. (PDF)

S3 Fig Edge boundary. The edge boundary of a retweet graph as the set of edges that connect a right-leaning cluster to a left-leaning cluster. A cluster is considered left-leaning if the average leaning of its users is less than –0.5 and right-leaning if the average leaning of its users is greater than 0.5. For each time window, we calculate the fraction of edges along the boundary that include at least one user belonging to an ideologically homogenous cluster. On average, across each time step in the Twitter data, only 2.87% of boundary edges contain a user from an IHC. S3 Fig. visualizes the retweet graph for the time window for November 3 through November 12, 2020, with clusters as each node.
In this graph, IHCs are highlighted in bright green, and edges are colored blue if their source node is left-leaning (red if their source node is right-leaning). The edges that make up the edge boundary are colored in purple. If edges in the graph were assigned randomly, we would expect 15.05% of boundary edges to contain users from an IHC. However, this graph has 3.08% of boundary edges including an IHC.
(TIFF)

S2 Table BookCorpus genres for labeling texts. Each literary genre included in our perplexity score labeling is described in S2 Table. Texts came from the BookCorpus dataset, which in turn scraped their data from the free e-book service Smashwords. On Smashwords, authors select their genres of their texts manually. This process leads to genres that are generally coherent but not necessarily in the manner that the genre title would imply.
The most impactful example of this phenomenon is the Futurism genre. Futurism is typically associated with writings that emphasize dynamism and technology, but in our data, the Futurism genre is used to label Christian apocalyptic fiction. This label is used consistently across texts, but does not match the traditional definition of futurism. For this reason, we change the Futurism label to the more accurate label Eschatology. Additionally, for some genres, the BookCorpus dataset is dominated by texts targeted at specific age groups. The Survival genre exclusively contains children’s stories, while the LGBT Fiction genre is exclusively young adult romance novels. For these reasons, we include descriptions of the genres rather than allow the genre titles to speak for themselves.
(PDF)

S4 Fig Facebook Genre Similarity Experiment. For our experiment, we calculate the average perplexity score for each of the 14 genres we investigated. The perplexity scores measure the word prediction entropy of a language model trained on writings from a certain genre; therefore, lower values of perplexity indicate that a text is stylistically closer to that genre. Each individual text was then given a binary label to signify whether it has a z-score of more than 1, meaning that the text is at least one standard deviation below this mean value. These binary labels are used as the input to a permutation test, the result of which tells whether the texts are closer to each genre than predicted by chance.
For the Facebook data, S4 Fig. visualizes the statistically significantly overrepresented genres for the anti-vaccine data and the pro-vaccine data. The expected value of 36% is displayed as a dotted vertical line. Anti-vaccine groups are visualized as red bars, and pro-vaccine groups are visualized as blue bars. The results show that the anti-vaccine groups are most similar to the speculative fiction genres and several hybrid genres. These genres, namely the Epiphanic and Eschatology genres, point to a personal narrative describing an awakening to the reality of corrupt activity hidden from view, and this is consistent with descriptions of conspiracy narratives. Comparatively, the pro-vaccine texts are most similar to the Dystopia speculative genre, the Political Treatises hybrid genre, and the Activism genre, a non-speculative genre indicative of more factual writing.
(TIFF)
