# Supplementary figures and images for: Identifying and characterizing ideologically homogeneous clusters on Twitter and Parler during the 2020 election
Source: PLoS One. 2025 Dec 10;20(12):e0338318. doi: 10.1371/journal.pone.0338318 (PMC12694848; doi:10.1371/journal.pone.0338318)

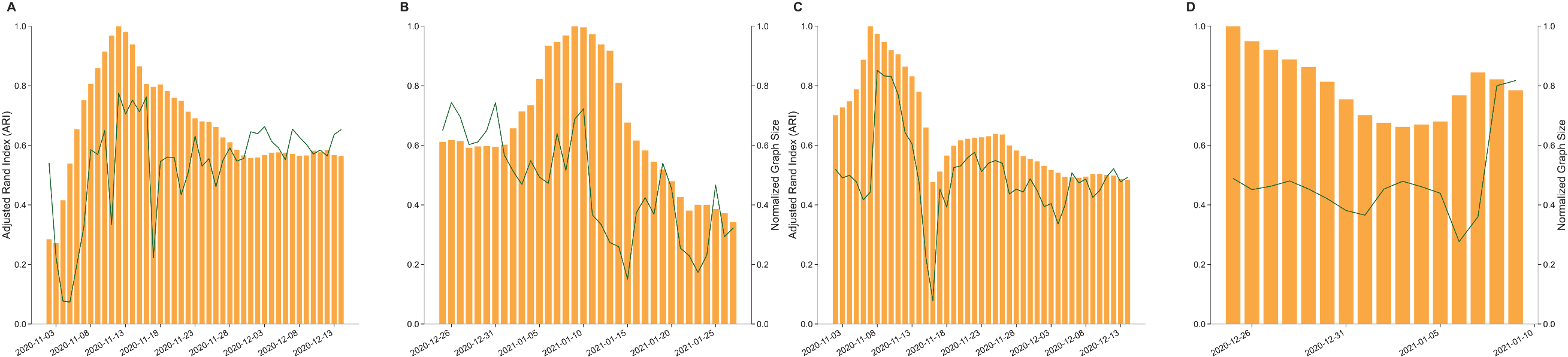

Supplement: S1 Fig — Before conducting experiments on our individual timestep clustering results, we must ensure that the clustering is stable over time. The clustering results could be dominated by noise and difficult to draw results from; in such a situation, a user’s neighborhood may be split into clusters randomly at each timestep rather than remaining stable. We measure this stability using the Adjusted Rand Index, which quantifies the number of pairs which are labeled consistently across two clustering results [60]. The Adjusted Rand Index (ARI) is calculated using: ARI=RI−E[RI]1−E[RI] (9) RI=a+bCn (10) where RI is the equation for unadjusted Rand Index, E[RI] is the expected value for the unadjusted Rand Index, a is the number of pairs that are in the same set in both clustering results, b is the number of pairs that are in different sets in both clustering results, and Cn is the total number of pairs in the data. The plots of ARI over time for Twitter are shown in S1 Fig. (A) and (B) and for Parler are shown in S1 Fig (C) and (D). In general, both plots demonstrate stable clustering over time, with slight daily noise caused by users joining the graph or genuinely moving clusters. An ARI of 0 signifies clustering results that are distributed uniformly at random, so both social networks having an ARI within the [0.4, 0.7] range means cluster stability is relatively high. Both platforms have a steep decline in ARI early on, which is attributable to a large number of new users entering the discussion. Twitter’s decline occurs within the first few days of the data, while Parler’s decline occurs closer to the date of the actual election; this is likely an artifact of Twitter being an established platform and Parler being a smaller platform reliant on new signups to grow its network. To visualize this relationship with the size of each day’s graph, the normalized size of each daily retweet graph is plotted alongside the ARI. After a long period of relative stability in both platforms, [file pone.0338318.s001.tif]

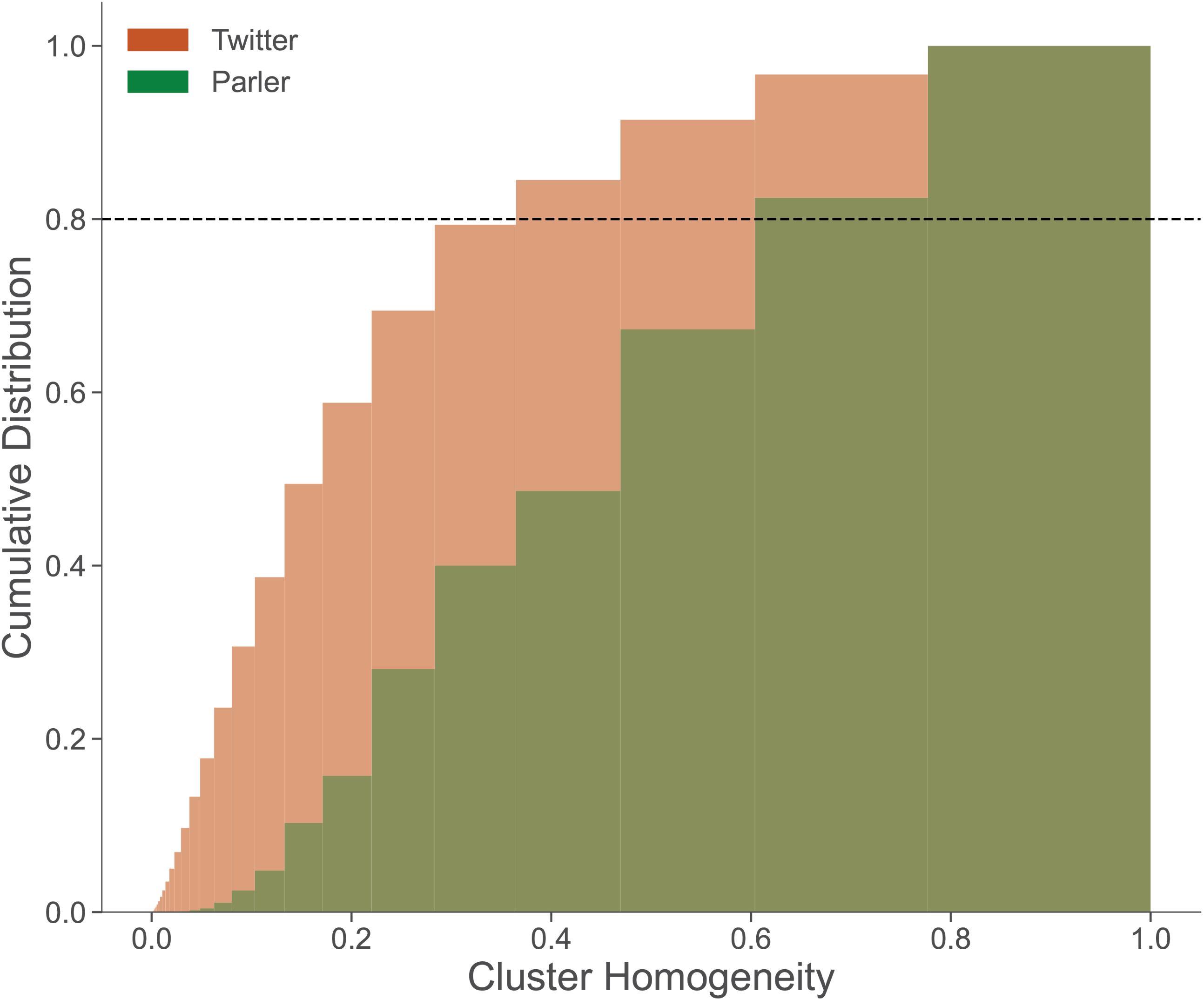

Supplement: S2 Fig — In the main text, we define IHCs as the top 20% of clusters according to the ideological homogeneity metric, and S2 Fig. visualizes the cumulative distribution of the ideological homogeneity metric for Twitter and Parler. The minimum value to be considered an IHC on Twitter is 0.375 (with an IHC mean of 0.607). To be considered an IHC on Parler, the minimum value is 0.75 (with an IHC mean of 0.892). In addition to showing that IHCs are more homogeneous on Parler than on Twitter, the distribution shows that clusters in general are more homogeneous on Parler than Twitter. The median ideological homogeneity on Twitter is approximately 0.2, while the median on Parler is approximately 0.5. The systemic differences between these two social media platforms, discussed in the main text, are the likely cause of these disparate distributions. (TIFF) [file pone.0338318.s002.tif]

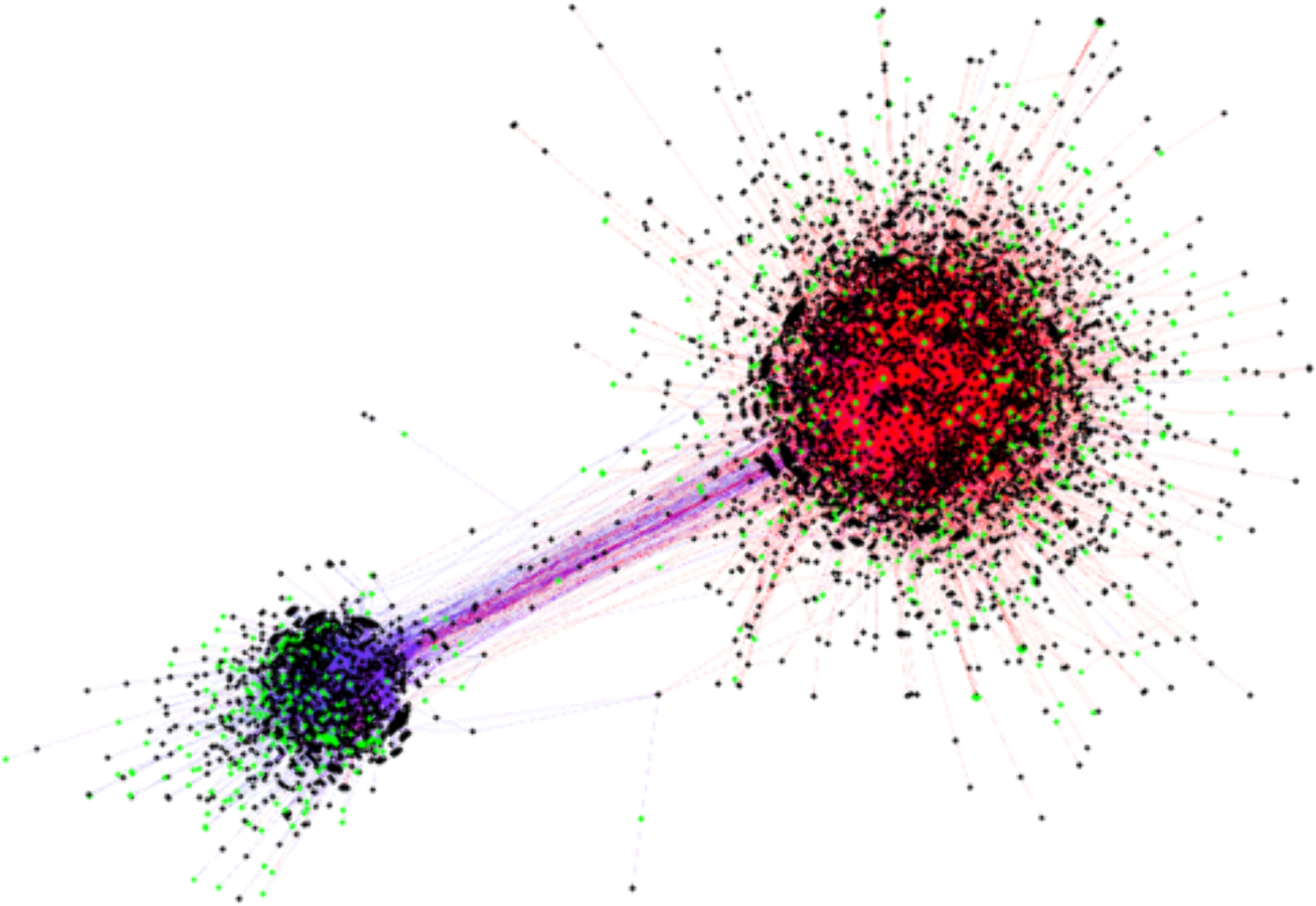

Supplement: S3 Fig — The edge boundary of a retweet graph as the set of edges that connect a right-leaning cluster to a left-leaning cluster. A cluster is considered left-leaning if the average leaning of its users is less than –0.5 and right-leaning if the average leaning of its users is greater than 0.5. For each time window, we calculate the fraction of edges along the boundary that include at least one user belonging to an ideologically homogenous cluster. On average, across each time step in the Twitter data, only 2.87% of boundary edges contain a user from an IHC. S3 Fig. visualizes the retweet graph for the time window for November 3 through November 12, 2020, with clusters as each node. In this graph, IHCs are highlighted in bright green, and edges are colored blue if their source node is left-leaning (red if their source node is right-leaning). The edges that make up the edge boundary are colored in purple. If edges in the graph were assigned randomly, we would expect 15.05% of boundary edges to contain users from an IHC. However, this graph has 3.08% of boundary edges including an IHC. (TIFF) [file pone.0338318.s004.tif]

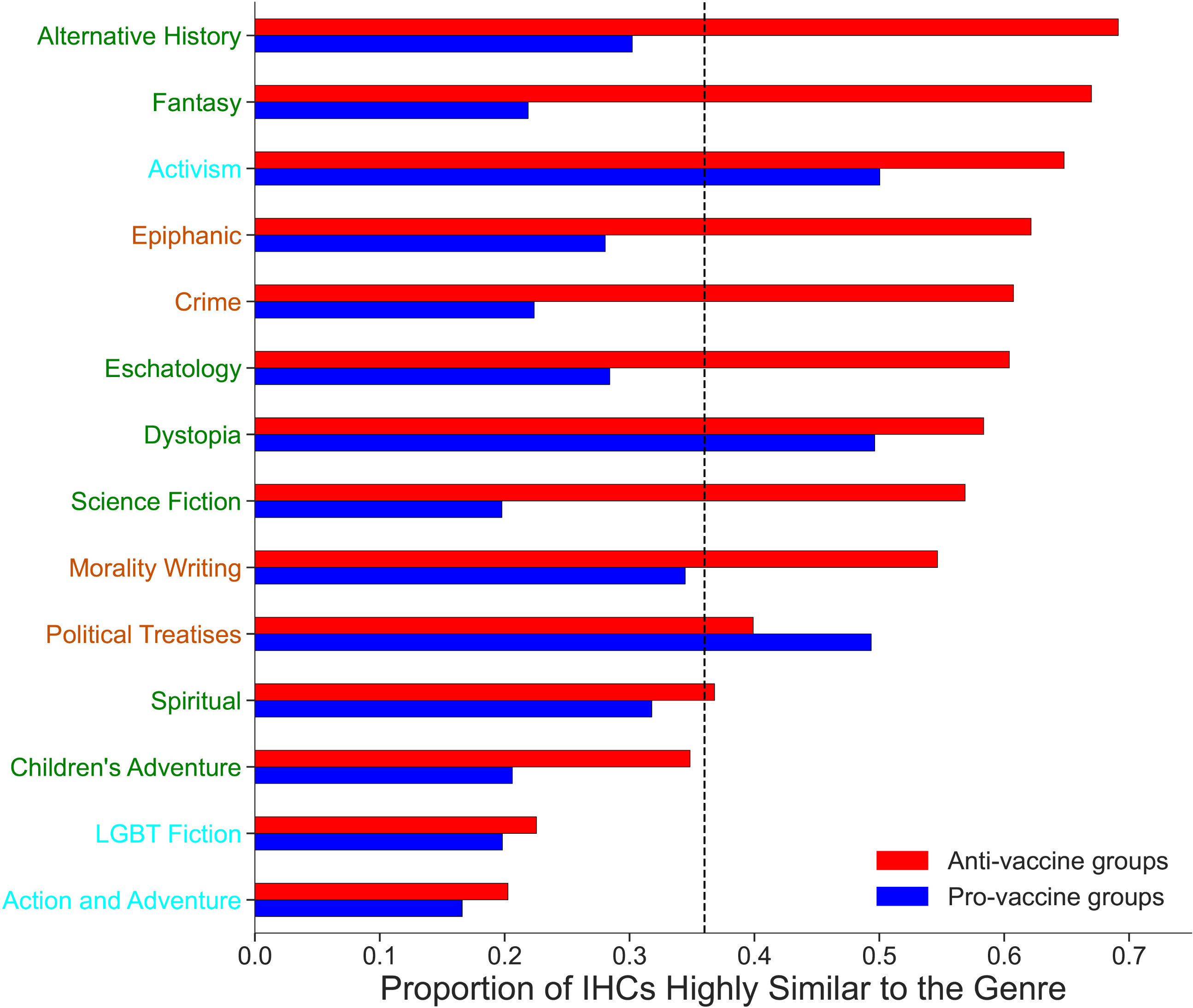

Supplement: S4 Fig — For our experiment, we calculate the average perplexity score for each of the 14 genres we investigated. The perplexity scores measure the word prediction entropy of a language model trained on writings from a certain genre; therefore, lower values of perplexity indicate that a text is stylistically closer to that genre. Each individual text was then given a binary label to signify whether it has a z-score of more than 1, meaning that the text is at least one standard deviation below this mean value. These binary labels are used as the input to a permutation test, the result of which tells whether the texts are closer to each genre than predicted by chance. For the Facebook data, S4 Fig. visualizes the statistically significantly overrepresented genres for the anti-vaccine data and the pro-vaccine data. The expected value of 36% is displayed as a dotted vertical line. Anti-vaccine groups are visualized as red bars, and pro-vaccine groups are visualized as blue bars. The results show that the anti-vaccine groups are most similar to the speculative fiction genres and several hybrid genres. These genres, namely the Epiphanic and Eschatology genres, point to a personal narrative describing an awakening to the reality of corrupt activity hidden from view, and this is consistent with descriptions of conspiracy narratives. Comparatively, the pro-vaccine texts are most similar to the Dystopia speculative genre, the Political Treatises hybrid genre, and the Activism genre, a non-speculative genre indicative of more factual writing. (TIFF) [file pone.0338318.s006.tif]
