# Supplementary material for: Identifying and characterizing ideologically homogeneous clusters on Twitter and Parler during the 2020 election
Source: PLoS One. 2025 Dec 10;20(12):e0338318. doi: 10.1371/journal.pone.0338318 (PMC12694848; doi:10.1371/journal.pone.0338318)
Supplement: S1 Table — (PDF) [file pone.0338318.s003.pdf]

S1 Table. Cluster bin sizes and counts

Cluster Sizes	Cluster Count
[3]	30800
[4,5]	29774
[6,7]	13110
[8,9]	8152
[10,12]	7968
[13,16]	6935
[17,22]	6918
[23,29]	5041
[30,39]	4677
[40,52]	3941
[53,69]	3262
[70,92]	2827
[93,122]	2325
[123,163]	2213
[164,217]	1806
[218,288]	1384
[289,384]	1160
[385,511]	912
[512,680]	693
[681,904]	469
[905,1203]	424
[1204,1601]	258
[1602,2130]	183
[2131,2833]	116
[2834,3770]	91
[3771,5015]	75
[5016,6672]	37
[6673,8876]	16
[8877,11809]	29
[11810,15710]	5
[15711,20900]	5
[20901,27805]	2
[27806,36991]	3
[36992,49212]	0
[49213,65469]	0
[65470,87098]	0
[87099,115872]	0
[115873,154151]	0
[154152,205077]	6
