# Supplementary material for: Identifying and characterizing ideologically homogeneous clusters on Twitter and Parler during the 2020 election
Source: PLoS One. 2025 Dec 10;20(12):e0338318. doi: 10.1371/journal.pone.0338318 (PMC12694848; doi:10.1371/journal.pone.0338318)
Supplement: S2 Table — Each literary genre included in our perplexity score labeling is described in S2 Table. Texts came from the BookCorpus dataset, which in turn scraped their data from the free e-book service Smashwords. On Smashwords, authors select their genres of their texts manually. This process leads to genres that are generally coherent but not necessarily in the manner that the genre title would imply. The most impactful example of this phenomenon is the Futurism genre. Futurism is typically associated with writings that emphasize dynamism and technology, but in our data, the Futurism genre is used to label Christian apocalyptic fiction. This label is used consistently across texts, but does not match the traditional definition of futurism. For this reason, we change the Futurism label to the more accurate label Eschatology. Additionally, for some genres, the BookCorpus dataset is dominated by texts targeted at specific age groups. The Survival genre exclusively contains children’s stories, while the LGBT Fiction genre is exclusively young adult romance novels. For these reasons, we include descriptions of the genres rather than allow the genre titles to speak for themselves. (PDF) [file pone.0338318.s005.pdf]

S2 Table. BookCorpus Genres for Labeling Texts

**Genre Title**	**Description**
Action and Adventure	texts across numerous genres (science fiction, children's, romance, Westerns, etc.) which heavily feature action-oriented scenes and plots rather than dialogue, exposition, or worldbuilding
Dystopia	texts set in a negatively-depicted futuristic (or near-future) world, usually ravaged by natural disaster, effects of hypercapitalism, or unchecked technology
Science Fiction	speculative fiction that builds a world around futuristic scientific and technological advances
Fantasy	speculative fiction which relies on non-scientific worldbuilding elements, such as magic and mythological creatures
Spiritual	evangelical texts which primarily serve to convert nonbelievers, primarily Christian and Muslim
Alternative History	speculative fiction in which historical events unfold differently than in reality
Epiphanic Writing	personal accounts of awakening moments and experiencing the universe as it truly is, largely from a New Age or Christian perspective
Morality Writing	a collection of primarily New Age and Christian texts which focus on on issues of morality in a spiritual context
LGBT	young adult stories that explore human sexuality, the internal process of understanding oneself, and the cultural process of 'coming out'
Political Treatises	collections of texts that express political commentary from several viewpoints
Eschatology	texts featuring a Christian apocalyptic or post-apocalyptic world, featuring accounts of the Rapture and the afterlife
Crime	largely nonfiction mixture of true crime, academic writing, and confessionals
Activism	nonfiction texts from multiple perspectives which promote awareness of social issues and suggest social reforms
Children's Adventure	texts written for middle-school or younger children which feature action-oriented stories, puzzles to solve, and occasionally fantastical elements
